# Beyond the plan: How land use control practices influence flood risk in Sekondi-Takoradi

**DOI:** 10.4102/jamba.v11i1.638

**Published:** 2019-05-06

**Authors:** Jerry C. Tasantab

**Affiliations:** 1 School of Architecture and Built Environment, University of Newcastle, Callaghan, Australia; 2 Kwame Nkrumah University of Science and Technology, Kumasi, Ghana

**Keywords:** land use, land use controls, flood risk, flood risk creation, vulnerability, Ghana, Sekondi-Takoradi

## Abstract

Using a pragmatic philosophical underpinning, this article analyses how land use planning actions and inactions contribute to flood risk creation in Sekondi-Takoradi, Ghana. In recent times, the planning system in Ghana has come under intense public criticism for failure to effectively control physical development in the major cities. The recurring flooding in the cities of Accra, Kumasi, Tamale and Sekondi-Takoradi seems to testify to this failure. Many lives and property have been lost through these flooding events in the country. This article argues from a disaster risk reduction point of view that the ineffectiveness in elementary processes of land use planning, such as delays in permit approval, inadequate monitoring and inspections, and lax enforcement of regulations, potentially creates flood risk. The rational of this study is therefore to bring to light the land use planning actions and inactions that create flood risk in Sekondi-Takoradi exposing urban dwellers to flooding. Because of the article’s pragmatic underpinning, a mixed-methods case study approach was adopted for this investigation. Both survey and interview data were collected from homeowners and planning institutions in Sekondi-Takoradi to ascertain how land use control practices seem to be contributing to flood risk in the municipality. The analyses comprised simple statistical analysis of the survey data in Statistical Package for the Social Sciences (SPSS) and thematic analyses of the interview data. The findings reveal that institutional incapacities have resulted in delays in permit approvals, non-compliance with permit regulations, uncontrolled conversion of vegetated land, lax monitoring and inspections of physical developments and poor enforcement. These institutional challenges have emboldened prospective land developers and homeowners to flout building and land use regulations. This has led to the building of residential properties in swamps, waterways and other flood-prone locations, creating flood risk.

## Background of study

In recent times, the planning system in Ghana has come under intense public criticism for failure to effectively control physical development in the major cities (Adarkwa 2012). The recurring flooding in the cities of Accra, Kumasi, Tamale and Sekondi-Takoradi seems to testify to this failure. Many lives and property have been lost through these flooding disasters in the country. On 03 June 2015, the most devastating flooding in the history of Ghana occurred in Accra, killing over 150 people.

A multiplicity of issues have been identified as causal agents of flood risk in Ghanaian cities, including uncontrolled urban land use development (Amoako & Inkoom 2017), rapid urbanisation, environmental degradation and worsening poverty conditions. These have overstretched the capacity of existing infrastructure, and exposed urban dwellers to environmental hazards particularly flooding (Korah & Cobbinah 2016). It has been argued that socio-economic pressures rather than ecological, health and safety, and environmental considerations have determined land uses resulting in limited regard to open spaces, areas liable to flooding and ecologically sensitive areas (Korah & Cobbinah 2016). Darkwah, Cobbinah and Anokye (2018) contest that urban planning in Ghana has failed to build resilient cities resulting in compounded stresses and shocks.

Notwithstanding the current nature of things, conscious efforts at ensuring harmonious land use planning and environmental sanity in Ghanaian urban settlements date back to the country’s era of colonialisation (Boamah, Gyimah & Bediako Nelson 2012; Tasantab 2016). Grant and Yankson (2003) attest that ‘zoning and building codes were strictly enforced to maintain an orderly character and environment in the planned district’, especially the Central Business District (CBD). This practice was to ensure that the growth of the cities promotes public health, orderliness and environmental sanity.

The country continued to rely on the provisions of this colonial era legislation, the Town and Country Planning Ordinance of 1945 (Cap 84) to ensure land use and development control until 2016 when a Land Use and Spatial Planning Law was passed by the Parliament of Ghana (Korah, Cobbinah & Nunbogu 2017). Until the Land Use and Spatial Planning Law, land use planning was guided by the Cap 84, the *Local Government Act* (Act 462, 1993), *Development Planning System (NDPS) Act* (Act 480, 1994), the *Environmental Protection Agency (EPA) Act* (Act 490, 1994) and the National Building Regulations (LI 1630, 1996) (Fuseini & Kemp 2015; Tasantab 2015). Even when Ghana’s decentralisation act, the *Local Government Act* (Act 462), was promulgated in 1993 and made stipulations for land use planning and physical development, the Cap 84 was not repealed (Tasantab 2015). Under these legal provisions, prospective developers were required to obtain development/building permit from relevant planning authorities.

These acts and regulations were meant to complement each other. Yet, their provisions were sometimes contrary. One contradiction that has seemingly facilitated illegal land use developments in flood-prone areas is found in the National Building Regulations (LI 1630, 1996). It states that when an applicant for development or building permit does not receive feedback on an application after 3 months, he or she can commence development with the assumption that the application has been accepted. This is despite the fact that most development applications for varied reasons are not granted after 3 months of submission. It has been found that between 2010 and 2013, only 41% of permit applications in Sekondi-Takoradi received grant notification after 3 months (Tasantab 2016). In addition to these, Ghana has a complex land management system. Ours is a dual land administration system where the majority (about 78%) of lands are under custodianship of chiefs and traditional leaders (Cobbinah & Aboagye 2017; Kleemann et al. 2017; Owusu-Ansah & Braimah 2013). This complex land management system ‘allows multiple traditional leaders to control and sometimes allocate land often contradicting planning requirements’ (Cobbinah & Aboagye 2017).

According to Adarkwa and Post (2001), the cities in Ghana have seen tremendous changes in size, density and areal extent. It is further observed that rapid urbanisation and development in Ghanaian cities have led to physical development problems, including:

- developments occurring in unapproved locations causing inconveniences
- incompatible land uses (Adarkwa & Post 2001; Boamah et al. 2012).

These problems persist notwithstanding the fact that these are major cities and considered to be planned. Land use controls are necessary for ensuring a safe living environment in urban areas (Boamah et al. 2012). It also ensures that physical development takes place in a manner that inures to the health and safety of its inhabitants (Tasantab 2016).

The persistence of these problems seems to suggest that current framework for land use planning has been ineffective (Adarkwa & Post 2001). The result of this is the recurrent flooding in the cities (Adarkwa 2012; Korah & Cobbinah 2016). Studies in Australia have observed that land use decisions can result in flooding of affected areas (Bird et al. 2013). The urban planning institutions in Ghana are considered weak, and planning officials are accused of granting permission to developers to construct homes in flood-prone areas (Korah & Cobbinah 2016).

This article therefore aims to investigate why land use control practices seem to be inadvertently creating flood risk in Sekondi-Takoradi, Ghana. The research question to be answered in this study is: in what ways do land use control practices influence flood risk in Sekondi-Takoradi?

### Urban flooding

Flooding is the overflow of water onto land surfaces that are normally dry, which may cause exceptional losses and destruction (Vojinović 2015). Heavy rainfall of long duration or of high intensity is one of the leading causes of flooding (Limthongsakul, Nitivattananon & Arifwidodo 2017). Surface water from this rainfall then builds up in areas of low elevation (Few 2013). Sudden but severe flash floods may also occur as a result of intense rainfall from rain storms and cyclones (Few 2013). Urban flooding takes different forms, including regular waterlogging of localised areas after rainfall (Houston et al. 2011). Although the waterlogging may be geographic, localised and remain for short durations, the devastation can be severe and hazardous (Few 2013; Limthongsakul et al. 2017). Flooding is usually severe in urban areas because of topography, loss of plant cover and vegetation, land use and obstruction of natural channels, and reduction in permeability of ground surfaces associated with urban development (Douglas et al. 2008).

Flooding has become a universally thorny issue (World Disasters Report 2016). The world is witnessing recurring episodes of flood hazards with succeeding events more severe than the previous (Douglas et al. 2008). Notwithstanding, it is anticipated that climate change will increase the proportion of annual precipitation classified as heavy floods (Collins et al. 2013; Erman et al. 2018; Kirtman et al. 2013; Wisner et al. 2015). This endangers the lives of the vulnerable who live in flood-prone urban areas (Oliver-Smith et al. 2016; Wisner et al. 2015).

Flood-related disasters are increasing globally (Brown et al. 2018; Wisner et al. 2015). This has resulted in the destruction of human lives and properties worth billions of dollars (Brown et al. 2018; World Disasters Report 2016). The World Disasters Report revealed that 1719 flood disasters occurred between 2006 and 2015. This resulted in the loss of 57 000 lives and 830 million affected people worldwide (World Disasters Report 2016). In monetary terms, the damage was estimated to be in excess of $342.7 billion between 2006 and 2015 (World Disasters Report 2016).

In Ghana, flooding is the most destructive natural hazard (Erman et al. 2018). The literature suggests that the International Disaster Database (EM-DAT) has recorded 3.9 million people directly affected by flooding in Ghana. The number of deaths as a result of these flood events has been estimated to be about 550 (including 03 June 2015 disaster). The economic damage exceeded $780 million (Asumadu-Sarkodie, Owusu & Rufangura 2015). In recent memory, the flood event that resulted in huge casualties occurred on 03 June 2015. A severe flood and a fire incident (attributed to the flood) in Accra resulted in the deaths of more than 152 people and several injured persons (Amoako & Inkoom 2017). Apart from these quantifiable data, the pain, trauma and health risk resulting from these disasters are hardly told (Revi et al. 2014).

### Disaster risk reduction through land use planning

Disaster risk reduction (DRR) is the concept and practice of reducing disaster (flood) risk through systematic efforts to analyse and manage the causal factors of disasters, including through reduced exposure to hazards, lessened vulnerability of people and property, wise management of land and the environment, and improved preparedness for adverse events (UN-HABITAT 2015). It focuses on the development and application of polices, strategies and practices to reduce vulnerabilities and disaster risk through prevention, mitigation and preparedness (Twigg 2004). Land use planning therefore has emerged as one of the ways to mitigate flood risk. It offers the opportunity to manage flood risk through its flexibility to address different types of floods, runoff, population growth and land cover changes. It also ensures ‘a safe, productive, and livable urban environment at lower cost as compared to using structural measures’ (Kryspin-Watson et al. 2017). Good practice land use planning enhances flood risk management (King et al. 2016). It therefore offers planners the opportunity to reduce flood risk and eliminate flood risk creation.

According to King et al. (2016), responsible land use planning can prevent or reduce the severity of impact that a natural hazard can have upon a community. On the contrary, irresponsible land use planning permits the development of settlements and infrastructure in locations that have a high exposure, susceptibility and likelihood of experiencing a natural disaster (King et al. 2016). Land use planning measures avoid and minimise flood losses by keeping developments away from floodwater through regulation of building design, sitting and building materials, and zoning. Hence it addresses existing and future risk (Jha, Bloch & Lamond 2012; Kryspin-Watson et al. 2017).

Land use planning has a pertinent role to play in flood (disaster) risk reduction by regulating the use of land in flood-prone areas (WMO 2016). It is imperative then to consider flood risk reduction and management at all stages of the planning process (Jha et al. 2012). Failure to ensure this seems to have led to ‘urban growth patterns that have significantly amplified the exposure of urban populations to flood risk’ (Bloch 2013; UN-HABITAT 2016).

### Study location

The city of Sekondi-Takoradi is the regional and principal commercial capital of the Western Region of Ghana and the Sekondi-Takoradi Metropolitan Assembly (STMA). The metropolis is located along the coast, about 280 km west of Accra and 130 km east of the Ghana-Ivory Coast border. The 2010 population and housing census found the population of the metropolis to be 525 614 (GSS 2014). The Sekondi-Takoradi city is now informally called the Oil City of Ghana because of the discovery and production of enormous oil resources in the Western Region (Tasantab 2015).

As the statutory authority that controls and promotes growth and physical development, the STMA issues development permits to prospective developers in the metropolis (Tasantab 2015). Sekondi-Takoradi was selected for this study because of its emerging prominence as a destination for oil-induced migrants from all parts of the country. Below is the location of Sekondi-Takoradi in the context of Ghana.

## Methods and data sources

Data for this article were obtained from a review of relevant literature and primary data collected in 2014. The review included extant literature on land use planning, flood risk and DRR. The review involved searches of databases such as Web of Science, Science Direct, Mendeley, Google Scholar and general Internet search engines.

The primary data were collected through face-to-face interviews and a survey in the STMA. The main aim of the 2014 data collection was to investigate the processes and practice of development or land use control in Sekondi-Takoradi. The objective was to investigate how it is done and the factors that promote or inhibit its effectiveness. By doing so, data on how people in Sekondi-Takoradi participate and/or patronise land use planning were obtained. This data was collected from homeowners and the land use planning institutions.

Semi-structured interview guides were used to collect data from key informants, while a survey instrument was used to collect data from the homeowners. The interviews were conducted with an official of the Town and Country Planning Department (TCPD), three officials from the Building Inspectorate Unit (BIU) of the STMA and a chief of one of the suburbs in Sekondi-Takoradi as participants. These key informants were experts in their fields and enabled the research to glean valuable knowledge from their expertise in land use management and planning.

Furthermore, the survey was administered to 400 homeowners (or their caretakers) in the Sekondi-Takoradi suburbs of Windy Ridge, Chapel Hill, Fijai, Adiembra and Keikuma. The sample size was determined using a sample size table available on the Internet at the confidence level and confidence interval of 95% and ±5, respectively. The sample communities were purposively selected for the sole purpose of focusing on the characteristics in Sekondi-Takoradi that would provide the best insight into the research problem. The research intended to capture common patterns (Palinkas et al. 2015) in land use planning that cut across communities in Sekondi-Takoradi. This helped to simplify analysis despite the multi-site case study.

**FIGURE 1:**
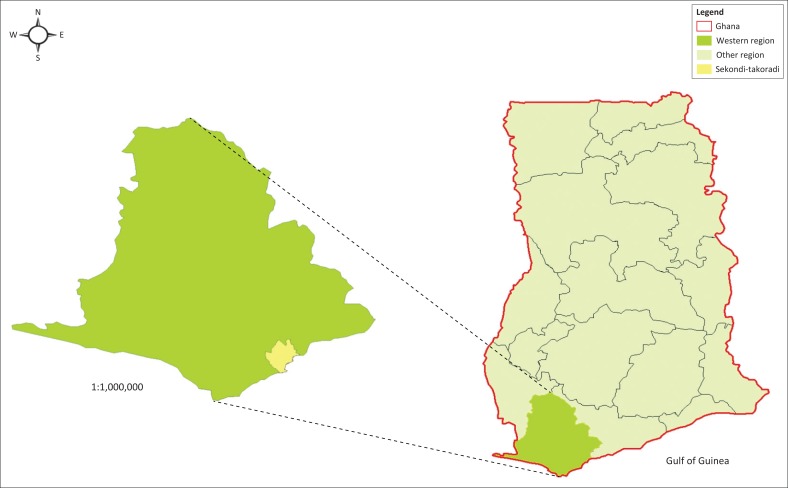
Context map of Sekondi-Takoradi Metropolitan Assembly in Ghana.

In each community, 80 surveys were conducted. This was because of the non-availability of clear population data for some of the suburbs sampled. Regarding the recruitment of homeowners for the household survey, a random starting point (mainly a prominent landmark like a church or chief palace) was determined. The survey team then followed routes in the settlement and picked houses to interview beginning from the random starting point. A selection interval of every fifth house was used. Because of considerations for availability and willingness to participate in the research (Palinkas et al. 2015), convenience methods were also used to select the houses in which to conduct the survey. Therefore, only house owners who were available at the time of the survey were included. For instance, if there were no occupants in the fifth house selected or the occupants were not willing to participate, it was replaced with the next house. At the beginning of each survey, verbal consent was obtained. The consent process made it explicit that participation in the study was voluntary and that the research was for academic purposes only. It also assured the participants of confidentiality. The participants were also assured that the final results of the study will be aggregated with others. Therefore, no personal identifying information would be included. Overall, the survey instrument included both close-ended and open-ended questions.

The interview data were recorded through audio recordings and notes. The audio recordings were later transcribed and synchronised with the notes. This was then formatted into themes and analysed accordingly. The survey responses, on the contrary, were recorded on the survey instruments and later transferred into Statistical Package for the Social Sciences (SPSS). A codebook was prepared of the survey responses. After each day, the entries on the survey instrument were vetted to check for entry errors and corrected accordingly. The data were then generated into simple frequency tables and charts. The following analysis is based on this data.

### Ethical considerations

The data for this article were obtained from a study conducted in 2014 in fulfilment of the award of MPhil Planning degree from the Kwame Nkrumah University of Science and Technology, Kumasi, Ghana. The process from the inception of the study to the data collection was reviewed by the teaching staff of the Department of Planning and verbal approval was given. Several presentations were made by the author after which the verbal approval was granted to commence the study. Compliance with ethical standards was therefore ensured.

## Findings

More than 80% of the disasters in Ghana are considered to be climate related (GoG 2013). It is expected that climate-related hazards, such as floods, will increase in frequency and magnitude (Brown et al. 2018; GoG 2013). The national climate change policy of Ghana therefore sought to ‘establish various measures to protect livelihoods and assets of vulnerable communities from climate-related risks’. It also sought to ‘strengthen the institutional framework for disaster risk response and management, and the capacities of agencies in disaster risk management’ (GoG 2013). Although the Ghanaian Government has implemented programmes such as public education of residents in flood-prone areas (Danso & Addo 2017) to reduce flood risk, risk are continuing to be created by land use practices. These are processes and practices that can render flood risk reduction programmes ineffective and defeat the objectives of the national climate change policy. One of these processes is the control of how land is used, what land is used, when land is used and the purpose for which it is used.

Land development in Ghana is expected by law to pass through several processes, including acquisition of land from chiefs, acquisition of land title documents from the Lands Commission, grant of building permit from the TCPD and inspection of building developments by the BIU. All these institutions and processes influence the nature of land use controls in Sekondi-Takoradi and Ghana at large. These processes and institutions also influence how land use planning reduces existing and expected flood risk. Findings from this study show that land use control processes and practices in the study area can create flood risk. These processes include grant of building permits and inspection or monitoring building developments in the study area.

### Development or building permits

Development and building permits serve as the primary means of implementing zoning and planning regulations to ensure that building developments are compliant. For this reason, considerable emphasis has been put on reforming the permitting system to reduce timelines and ensure timely issuance of permits. This was one of the purposes of the Land Use Planning and Management Project (commencing in 2007) to reform land use planning and management in Ghana (Fuseini & Kemp 2015). The issuance of building or development permits is usually a long process, and requires the prospective developers to provide several documents to verify the ownership of the land and compliance with the local plan of the area. This process can therefore be used to ensure that prospective developments are restricted from flood-prone areas. However, the following findings illustrate how it rather creates flood risk.

#### Delay in permit approval

Data from the TCPD revealed that 58.7% of residents who lodged permit applications (between 2010 and 2013) did not receive information on the approval of their applications after 3 months. The 3 months period is referenced because the National Building Regulations in Ghana (LI 1630) stipulate that prospective builders can go ahead to build after 3 months if they do not receive information on whether permits have been granted or refused. However, these delays are often such that respondents do not receive their permit decisions after 3 months. Although 51% (206) of our respondents initiated and completed the construction of their dwelling homes, about 64% of these reported delays of more than 3 months before they received their permit decisions. On the contrary, only 22.8% of respondents received their permit approval within 1–3 months duration. The rest (13.1%) could not remember how long it took to obtain their permits.

Some of the aforementioned permit applicants therefore took advantage of those delays and the loopholes in the National Building Regulations to build their homes in flood-prone locations. It is little surprising that Danso and Addo (2017) found that residents of Sekondi-Takoradi have to repeatedly cope with flood risks in the rainy season.

Although the TCPD recognised the negative implications of these delays, they cited logistical and resource constraints. This supports observations of (Adarkwa 2012; Cobbinah & Aboagye 2017) that the Planning Departments in Ghana are made ineffective by obvious limitations of logistics and manpower. The TCPD had only one planner for the entire metropolis. They, however, required five officials. Faced by many competing demands, the results are the delays in granting permits. Indeed, it has been theorised that Ghana has short supply of spatial planners and the few in service are gradually retiring (Yeboah & Obeng-Odoom 2010). This makes the TCPDs understaffed and unable to reliably perform their duty of controlling land use developments. In terms of logistics, the department had one vehicle for both field inspections and other official duties.

These factors were found to have hampered the process of land use controls in the municipality. These shortcomings conspired with other factors, such as high demand for land, to allow areas that were clearly prone to flooding to be built-up. Documents supplied by the department also revealed that there is intense pressure on housing, with about 40% of the population living in unacceptable housing conditions. This pressure coupled with the delays in the permitting process includes obvious factors contributing to housing development in flood risk zones. This situation has led to unsustainable land development (Kleemann et al. 2017; UN-HABITAT 2016), which the Government of Ghana seeks to achieve by becoming a signatory to the Sustainable Developments Goals.

#### Non-compliance with permit regulations

Studies have shown that permit regulations in Ghana are usually not complied with by developers. In Sekondi-Takoradi, 38% of buildings were found to have been constructed without permits (Somiah, Ayarkwa & Agyekum 2015). The present study confirms this to be the case. Observations show that buildings were constructed in areas prone to flooding or demarcated as flood-prone areas in the development plans of the metropolis. Several factors, including delays in approval of building permits, inadequate public awareness of the requirements for building permits or the notion that building permits are unnecessary, were given by respondents to explain this lack of compliance.

#### Uncontrolled conversion of vegetated land

According to documentary evidence received from the TCPD during the survey, 6% of lands that were previously demarcated as green spaces or farmlands have been converted into residential uses. This is supported by Kleemann et al. (2017) whose remote sensing analysis revealed a 7.1% increase in the built-up area between 2007 and 2013. This obviously has the potential of increasing the risk of flooding in the area. In their guide on Forensic Investigations of Disasters (FORIN), Oliver-Smith et al. (2016) made the link between land conversion from vegetation cover to developed land and flood risk. They reported that more than 50% of the area of Metro Manila was covered with vegetation, and by 2009 more than 80% of such land had been converted to impermeable built-up areas, resulting in narrowing of river channels, lost estuaries and waterways, and the siltation of rivers and land subsidence. This greatly increased the potential for flooding (Oliver-Smith et al. 2016).

### Inspection of on-going land use developments and monitoring

Apart from the permitting processes, inspection and monitoring of physical development is also important for ensuring compliance with development plans and regulations. In Ghana, the responsibility for this according to the *Local Government Act* (Act 462) and the National Building Regulations (LI 1630) is the purview of the BIU. This unit is part of the Works Department of the Assembly, and actively participates in the permit approval process. The unit is also supposed to ensure that physical developments comply with land use plans and zoning regulations of the metropolis. The unit is further responsible for implementing the requirements and conditions of the issued building or development permits. This unit therefore plays an active role in ensuring the success of land use controls and compliance with land use regulations in the metropolis. However, the following findings explain why the current practices of the unit may be contributing to flood risk creation.

#### Lax inspection and monitoring of physical developments

This is another issue which can potentially create the opportunity for prospective land developers and chiefs to circumvent land regulations to encroach on flood-prone land. Officials of the BIU emphasised that:

‘we are supposed to be on the field. We go for field visits, if we find any site cleared, we start monitoring the area. When we see any structure coming up, we go and ask for the permit and from this time, monitor the area always to ensure that the development is according to the planning scheme.’ (Mr Kofi Kojo, Survey and Mapping Officer, Building Inspectorate Unit)

The above statement on face value suggests that the building inspectors are doing their job. It, however, reveals the reactive nature of their endeavour.

In addition, only the physical developments spotted by the building inspectors are brought under the demands of law. Those not seen by the inspectors continue to develop, even if they do so without permits. Also, there is no initiative in the study area that encourages residents to report such developments to the planning authorities. In the absence of these checks, people embark on unauthorised developments. The BIU respondents further stated that ‘We are not able to monitor all projects. The reason is that we do not have a car to be able to go for field inspections’. Indeed, this inability on the part of these officials to monitor developments can directly be attributed for the high rate of non-compliance with building regulations, not only in Sekondi-Takoradi but also in Ghana as a whole. A study by Baffour Awuah and Hammond (2014) in Kwabenya, Accra, found a non-compliance rate among residential developments to be as high as 93%.

#### Poor enforcement

Apart from monitoring and inspection of developments, enforcement is also key to ensure compliance. Enforcement is required to deal with cases in which developments are carried out either without planning permission or in breach of the conditions or limitations attached to a grant of planning permission. It is also required to ensure that developments are carried out according to the local plan of an area. Enforcement as a tool for ensuring compliance with planning regulations hinges upon inspections and monitoring of development activities.

According to the BIU official:

‘When we visit any site and the developer fails to produce a permit, we issue a stop work notice. When the person fails to comply with the stop work notice, he/she is issued an enforcement notice. The stop work notice usually lasts for a week after which an enforcement notice is issued for a week if the developer fails to adhere to the stop notice. When both notices are ignored, we proceed to court to obtain a demolishing warrant.’ (Mr Kofi Kojo, Survey and Mapping Officer, Building Inspectorate Unit)

This narrative clearly portrays that developers do not often comply with building regulations. The key informants further stated:

‘A developer who acts upon the notices is made to go through the normal process of obtaining a permit. However, the person is made to pay a fine in addition to the permit fees. But if the on-going development does not conform to the original use to which the site was zoned, the developer is made to apply for change of use. When this is granted, the developer can go ahead to develop the property.’ (Mr Kofi Kojo, Survey and Mapping Officer, Building Inspectorate Unit)

Several concerns surface immediately from this narrative. Why would a developer who has clearly violated planning regulations be given the opportunity to have an area rezoned to accommodate his or her development? This is most concerning because developers can deliberately flout planning and building regulations, knowing full well that afterwards their development would be approved when they apply for change of use or rezoning. While changing the use of land from residential or commercial to recreational or green spaces can reduce flood risk (Bird et al. 2013). The above practice rather does the opposite. The issue of fines was also noted to facilitate non-compliance with regulations. It was found that construction normally continues after the penalties are paid. Although the developers assume that penalty is a payment for the building permit, the inspectors relax their inspections because such offenders have already paid fines (Yeboah & Obeng-Odoom 2010). As a consequence of the inability of officials to monitor development and the non-compliance of developers with land use regulations, housing development is allowed to take place in flood risk zones in the study area.

## Discussion

In answering our research question, in what ways do land use control practices influence flood risk in Sekondi-Takoradi, the article tried to portray how land use practices (actions and inactions) create flood risk. The study depicts that when land use controls are unable to properly order the right use of land, individual house developers will make decisions about the use of land that create flood risk. It is posited that risk is the combination of the probability of a hazard event occurring and its negative consequences (UNISDR 2009). Therefore, land use control processes or practices (actions and inactions) that situate people in locations that would bring negative impacts when flood hazard events occur are creating risk.

The presence of risk (specific physical and social conditions) directly influences the occurrence of disasters (Lavell et al. 2012). Land use is a component of the broader social, economic, environmental and physical processes shaping risk (Romero-Lankao et al. 2014). It is therefore important to understand how the interaction of hazards and vulnerabilities takes place at the local level (families, communities and individual buildings) (Lavell et al. 2012). More people in urban areas are exposed to flood risks because the process of urbanisation has amplified failures in the land use planning regime as our findings show (Oliver-Smith et al. 2016; Wisner et al. 2015).

Our findings portray that land use control processes and practices are in a sense allowing housing developments to take place in floodable areas. This has been revealed to be a result of lack of staffing and logistical capacity to effectively control land use development in the area. The task of ensuring that every land use in Sekondi-Takoradi ensures the health and safety of lives and property seems to be under-resourced. This allows housing developments in flood-prone areas. Thus, any sustained precipitation results in flooding as identified by Amoako and Inkoom (2017) and others (Amoako & Frimpong Boamah 2014; Erman et al. 2018; Rain et al. 2011). This is worrying because heavy precipitation is anticipated to increase in Ghana over the period 2010 and 2050 (World Bank & GFDRR 2011).

Although there have been attempts to reform the land use planning system through the Land Use Planning and Management Project (2007–2010) to make it more efficient, the study area being one of the pilot locations, the bottlenecks persist. Flood risk is created when populations are located in flood-prone areas, creating the possibility for property and lives to be lost when extreme flooding occurs (Raaijmakers, Krywkow & van der Veen 2008). Although there are many measures such as embankments and dikes to defend against floods, land use planning is considered a viable compliment to these measures. This is because of its ability to remove developments from the floodable areas through zoning. However, this is not being done in the study area because of the aforementioned findings. There are pressures for urban development to extend into areas with higher flood risk (COAG 2011). If executed properly, land use control can be the first line of defence to limit exposure to these risks (Wilby & Keenan 2012).

## Conclusion

In conclusion, if land use planning is to be a potent flood risk reduction and mitigation measure in Ghana, then efforts must be made by the Government of Ghana, and land use and management institutions such as the Town and Country Planning Authority and BIU to address the challenges revealed in this study. Therefore, permit approval times should be improved so that most permits are approved within the stipulated 3 months. It is also important to improve the inspection and monitoring of the entire metropolis to enforce permit conditions and building regulations and ensure compliance. The land use planning institutions should also actively engage the chiefs and citizens of the metropolis and encourage them to report all physical development taking place in their locality. This will enable the departments to be up-to-date about developments in the communities. Compliance with land use regulations will be improved if enforcement is pursued rigorously. With the current resource and staffing constraints hampering every aspect of land use planning in Sekondi-Takoradi, it is highly imperative for the government to give prominent attention to resourcing these departments. It is only then that they can effectively execute their functions.

Land use planning or control is an essential aspect of flood risk reduction. Planning in Sekond-Takoradi should therefore ensure that development occurs in areas where risk is less likely to be created. ‘If there are no people or buildings in areas prone to floods, then there will be no risk’ (Maier, Riddell & van Delden 2017).
